# TSPO PET Imaging: From Microglial Activation to Peripheral Sterile Inflammatory Diseases?

**DOI:** 10.1155/2017/6592139

**Published:** 2017-09-25

**Authors:** Bérenger Largeau, Anne-Claire Dupont, Denis Guilloteau, Maria-João Santiago-Ribeiro, Nicolas Arlicot

**Affiliations:** ^1^CHRU Tours, 2 Boulevard Tonnellé, 37044 Tours, France; ^2^Institut National de la Santé et de la Recherche Médicale U930, 10 Boulevard Tonnellé, 37032 Tours, France

## Abstract

Peripheral sterile inflammatory diseases (PSIDs) are a heterogeneous group of disorders that gathers several chronic insults involving the cardiovascular, respiratory, gastrointestinal, or musculoskeletal system and wherein inflammation is the cornerstone of the pathophysiology. In PSID, timely characterization and localization of inflammatory* foci* are crucial for an adequate care for patients. In brain diseases,* in vivo* positron emission tomography (PET) exploration of inflammation has matured over the last 20 years, through the development of radiopharmaceuticals targeting the translocator protein-18 kDa (TSPO) as molecular biomarkers of activated microglia. Recently, TSPO has been introduced as a possible molecular target for PSIDs PET imaging, making this protein a potential biomarker to address disease heterogeneity, to assist in patient stratification, and to contribute to predicting treatment response. In this review, we summarized the major research advances recently made in the field of TSPO PET imaging in PSIDs. Promising preliminary results have been reported in bowel, cardiovascular, and rheumatic inflammatory diseases, consolidated by preclinical studies. Limitations of TSPO PET imaging in PSIDs, regarding both its large expression in healthy peripheral tissues, unlike in central nervous system, and the production of peripheral radiolabeled metabolites, are also discussed, regarding their possible consequences on TSPO PET signal's quantification.

## 1. The Sterile Inflammatory Response and PET Imaging of Inflammation

Inflammation and its protagonist, inflammatory cells, act as the initial host defense to struggle against infection and injury. Immune system diseases can be dichotomized into autoimmune disorders, in which several actors lead to intrinsic hyperactivity of sensor/pattern-recognition receptor function, causing exacerbate and dysregulated immune response [1] and immunodeficiency diseases (i.e., inherited/primary or acquired/secondary), characterized by an inability of immune system to contain infectious disease and cancer development [2]. In both cases, the inflammatory processes are unappropriated; this explains the paradoxical relationship between immunodeficiency diseases and autoimmunity [3]. In inflammatory conditions, following exposure to aseptic stimulus involving physical chemical or metabolic signal such as burns, trauma, and dead cells, a cascade of response will be initiated by the release of local chemokines, interleukins, and prostaglandins, which are well-known proinflammatory mediators [4, 5]. Monocytes, macrophages, dendritic cells, and neutrophils are first-line immune effectors located in the interface between innate and adaptive immunity. Apart from autoimmunity disorders, noninfectious/sterile inflammatory diseases include various conditions where the leading cause of inflammation is acute and/or chronic exposure to irritant particles [5]. These sterile stimuli are different in nature and can be induced by drug therapy [6], alcohol consumption [7], exogenous particulates such as silica dioxide [8], asbestos [8], cigarette smoke [9], or endogenous particulates as well as monosodium urate [10], amyloid- *β* [11], and cholesterol [12]. Macrophages are a key player in the pathophysiology of sterile etiology, autoimmunity disorders [13], and irritant particles mediated diseases [5]. Macrophage activation starts with a proinflammatory phase, called M1 state. Sterile injuries provoke inflammation similar to injury caused by pathogens but M1b macrophage phenotype predominantly develop, before being polarized into M2 phenotype which induce anti-inflammatory/profibrotic response [14]. Given the fact that macrophages play a crucial role in sterile inflammatory processes, macrophage imaging appears to be a promising approach to assess, better characterize, and improve the diagnosis of disorders related to sterile inflammation.

Diagnosis of most peripheral sterile inflammatory diseases (PSIDs) is based on history, clinical symptoms, biology, serology, and conventional imaging technique such as radiological analysis or magnetic resonance imaging (MRI). Concerning its signs and clinical features, the inflammatory process is very stereotypic and nonspecific [5] to such an extent that the only symptoms can be asthenia, arthralgia, and fever. Furthermore, in front of inflammatory syndrome, clinicians have to make a decision about an infectious or sterile etiology and, sometimes, practitioners in internal medicine have recourse to trial corticosteroid therapy or empiric antibiotic therapy to statute despite the risk of complicating ulterior diagnosis [15]. Indeed, corticosteroids or anti-infectious drug could conceal the pathogenic process and therefore make uninformative conventional imaging techniques due to a lack of sensitivity, so the residual disease remains inaccessible. Similarly, inflammatory* foci* cannot be detected in the early phase of development because of the lack of substantial anatomical changes at this time. In front of several conventional exam limitations (e.g., invasive, lack of repeatability) and in order to investigate PSIDs by a deeper approach and to access pathophysiology, the use of molecular imaging and especially PET imaging has increased significantly in recent years. PET is a molecular and functional imaging modality, which permits repeated and noninvasive determination and quantification of specific biological and pharmacological processes [16] whose interest is in early diagnosis and to monitor/follow up the residual disease is promising. ^18^F-FDG represents the most widely used PET tracer and many authors have elucidated in detail relationship between inflammatory response, local hyperaemia, and hypervascularisation and uptake of ^18^F-FDG [17, 18]. Various PSIDs have been investigated with ^18^F-FDG including atherosclerosis [19], vasculitis [20], valvular inflammation [21], myocardial inflammation [22], rheumatoid arthritis [23], and Crohn's disease [24]. Nevertheless, ^18^F-FDG is a glucose analog which indicates an increase of glucose consumption, which in itself can be indicative of other on-going processes such as cancer, cell regeneration, or muscle contraction as seen in peristalsis. Furthermore, another limitation concerning its pharmacokinetic, the renal pathway by which ^18^F-FDG is eliminated, obstructs the image quantification [25].

Choline as an important precursor of membrane phospholipids has been labelled to image inflammatory diseases such as atherosclerosis [26–28] with a greater sensitivity in detecting atherosclerotic plaques than ^18^F-FDG [28]. Besides metabolic PET tracers of inflammatory cells (i.e., ^18^F-FDG and ^18^F-Choline), radioligands have been developed to evaluate more accurately peripheral sterile inflammatory processes. Among these, membrane receptor such as 18 kDa translocator protein (TSPO) and B lymphocyte CD20 antigen [29], cytokines like cyclooxygenase subtype 2 [30], matrix metalloproteinase [31], interleukin-2 [32] or endothelial adhesion proteins such integrin *α*v*β*3 [33], vascular adhesion protein-1 [34], and vascular cell adhesion molecule-1 [35] have been targeted. In this review, we focus on the most recent preclinical and clinical applications of TSPO PET imaging in PSIDs and discuss the potential added value in the clinical practice.

## 2. TSPO PET Tracers

TSPO is a highly hydrophobic five-transmembrane domain protein mainly situated in the outer mitochondrial membrane and is highly expressed in macrophages [36, 37]. TSPO is widely distributed in most peripheral organs including kidneys, nasal epithelium, adrenal glands, lungs, and heart, whilst the highest concentrations are in the steroid producing tissues, and is also minimally expressed in resting microglial cells in the healthy brain [38]. Numerous TSPO PET tracers have been developed and used mainly for the imaging of neuroinflammation [39].

In addition to many endogenous compounds like cholesterol or porphyrin, TSPO binds a range of synthetic ligands. Historically, the benzodiazepine ^11^C-Ro5-4864 was the first ligand able to discriminate peripheral from central benzodiazepine receptors [40]. Over the past few years, several structure activity relationship studies have established that four main domains are necessary for a ligand to interact with the TSPO, three major lipophilic regions, and one hydrogen-bond donor group [41]. Thus, several classes of TSPO radioligands have been synthesized, with, for most of them, the availability of compounds radiolabelled with carbon-1 (^11^C) or fluorine-18 (^18^F). The most widely used TSPO PET radiopharmaceutical, namely, ^11^C-(R)-PK11195, an isoquinoline carboxamide developed in the early 1980s, was the first nonbenzodiazepine type compound identified to bind to TSPO with high affinity (human dissociation constant,* Kd* = 2 nM) [42]. Despite having been tagged as the gold standard of TSPO ligands, ^11^C-(R)-PK11195 has several drawbacks including the short half-life of carbon-11, a low brain bioavailability, and a poor signal-to-noise ratio due to high nonspecific binding [43]. To counteract these limitations, there has been a great effort for the development of second-generation TSPO PET radiotracers. Among them, the derivatives of phenoxyarylacetamides (^11^C-DAA1106, ^11^C-PBR28, ^11^C-PBR06, and ^18^F-FEPPA) [44–47], designed from a chemical structure based on the opening of the diazepine ring of Ro5-4864, the derivatives of imidazopyridines (^11^C-CLINME), and the derivatives of pyrazolopyrimidines (^18^F-DPA-714) [48] are included.

Furthermore, a single nucleotide polymorphism in the TSPO gene (rs6971) leading to an amino-acid substitution (A147T) has been identified [49]. This polymorphism affects the binding affinity properties of the most of TSPO with a huge heterogeneity in PET images and their associated quantitative data. Three distinct binder statuses have been identified: HAB, high- (A/A; ~70%), MAB, mixed- (A/T; ~21%), and LAB, low-affinity binders (T/T; ~9%) [50]. The fact that the second-generation radiopharmaceuticals have been found to be sensitive to this polymorphism leads searchers to develop rs6971-insensitive radioligands. For this purpose, ^18^F-GE180, a third-generation TSPO tracers, was evaluated and seemed to be less sensitive to the TSPO-binding affinity status than the second generation [51]. Unfortunately, the small sample size and the exclusion of the LAB from this preliminary study leaded do not allow concluding that ^18^F-GE180 is insensitive to the polymorphism. Then, a new analog of ^11^C-(R)-PK11195, called ^11^C-ER176, showed little sensitivity to rs6971 when tested in vitro and had high specific binding in monkey brain [52]. Nonetheless, the clinical relevance of this third-generation compound remains to be confirmed.

## 3. TSPO PET Imaging of PSIDs

### 3.1. Inflammatory Bowel Diseases (See Table 1)

Although the etiology of inflammatory bowel diseases (IBDs), including Crohn's disease (CD) and ulcerative colitis (UC), is still unclear, it is widely accepted that both result from a mucosal immune response dysregulation in genetically predisposed individuals, for which susceptibility to IBD is mainly determined by complex interactions of environmental and genetic factors [53–55]. IBDs are characterized by various aspects of the inflammatory response, ranging from acute to chronic stages, and, in many cases, describe a cyclic evolution where attacks are interspersed with gastrointestinal stable periods. The natural history of the IBDs usually progresses to include strictures, fistulas, and abscesses [56]. Whereas in CD the whole gastrointestinal tract can be affected even if there is a predilection for ileocolonic involvement, UC is reduced to the caecum, colon, and rectum [57]. Localizing accurately the inflamed tissues is one criteria permitting differential diagnosis between the different types of IBDs and other diseases [58, 59]. Presently, the diagnostic approach relies on various exams ranging from clinical symptoms, such as bloody diarrhea, abdominal pain, asthenia, and weight loss, to serology, endoscopic exploration, radiological analysis, and nuclear medical investigations [25]. Nevertheless, 10 to 15% of all colorectal IBDs cases cannot be classified as UC or CD [60]. In order to provide information concerning the inflammatory processes in IBD, ^18^F-FDG PET imaging has been used in preclinical studies [25, 61, 62] and clinical studies [61, 63–67], especially in pediatric populations [64, 65, 68], and exhibited good diagnostic performances in patients with suspected IBD [58, 69]. Despite the well-known TSPO overexpression in colon cancer [70] and the knowledge that TSPO regulates the growth of colorectal cancer cells [71] which is also an unfavorable prognostic factor in stage III colorectal cancer [72], the involvement of TSPO in IBDs and dysplasia has not yet been completely investigated. After 7 days of treatment by dextran sodium sulfate (DSS), in a way to induce IBD in a rat model, Ostuni and colleagues [73] reported a TSPO overexpression, assessed by immunohistochemistry, in the rat colon of treated animals. In DSS model, whereas the increase of ^18^F-FDG colon uptake did not reach a significance level, ^18^F-DPA-714 colon binding showed a significant increase, compared to healthy animals. In addition, the relationship between tissue expression of TSPO, objectified by immunohistochemistry, and ^18^F-DPA-714 uptake provides the proof-of-concept that TSPO PET radioligand can be used to evaluate dynamically peripheral inflammation [25]. These findings lead us to think that TSPO PET imaging could serve as a more precocious biomarker than ^18^F-FDG to highlight the inflammatory processes of IBDs. Regarding the inflammatory cascade of IBDs, a recent study established the responsibility of the interleukin-33 (IL-33) pathway by modulating macrophages phenotype in IBDs [74]. Indeed, the authors demonstrated that IL-33 is involved in M2 macrophage polarization in inflamed mucosal samples from patients with IBD. Moreover, serum IL-33 levels were significantly lower in IBD's patients than those in healthy volunteers. Serum soluble suppression of tumorigenicity-2 (sST2) levels and its soluble receptor were significantly correlated with the pMayo score in UC patients but not in CD, supporting evidences that UC is a disorder linked to Th2-hyperpolarization in contrast to CD, which is rather Th1-derived [75].

### 3.2. Liver Diseases (See Table 2)

The expression of TSPO in the liver of healthy humans is usually low [76, 77]. Recent evidences suggest that TSPO might be contribute to the liver damage and alcoholic hepatitis progression. Indeed, TSPO gene is upregulated in alcohol hepatitis and appears to be involved in various biological processes which are determinant in the pathophysiology of alcoholic liver disease such as regulation of reactive oxygen species, response to alcohol, negative regulation of nitric oxide pathway, and regulation of necrotic cell death [78]. Tissue analysis revealed more serious steatotic aggregates and necroinflammatory infiltration in the higher uptake region of ^18^F-FEDAC. Autoradiography experiments [79, 80] supported* in vivo* PET data where TSPO tracer binding was not homogeneously distributed in the livers with nonalcoholic fatty liver disease (NAFLD) [79] and in the fibrotic livers (induced by carbon tetrachloride treatment) [80]. Analysis association between ^18^F-FEDAC binding and NAFLD activity score is in consonance with* in vitro* findings where TSPO overexpression was reported in activated hepatic stellate cells [81] and in the adipocytes of stressed rats with adipocytes aggregates and neoangiogenesis process [82]. TSPO PET tracer uptake increased in parallel with liver disease severity whether it is in NAFLD [79] or in fibrosis liver models [80]. Positively correlation between hepatic TSPO and (transforming growth factor) TGF-*β*1/(tumor necrosis factor) TNF-*α* mRNA expression [80] suggests that macrophage M1 and M2 phenotypes intervene during liver fibrosis development [83]. Complementary clinical studies with various liver diseases characterized by inflammatory processes (e.g., viral hepatitis, cholestatic hepatitis, autoimmune hepatitis, and Wilson's disease) are required to evaluate the ability of differential diagnosis of TSPO PET imaging.

### 3.3. Inflammatory Lung Diseases (See Table 3)

In contrast to nonspecific current methods (e.g., chest radiography and computed tomography), TSPO PET imaging could potentially give quantitative information about macrophage trafficking and kinetics, in order to evaluate treatment response and contribute to our understanding of the pathophysiology of lung noninfectious inflammatory processes [84]. Moreover, invasive methods (e.g., lung biopsy and bronchoalveolar lavage) are unacceptable for repeat measurement in the context of longitudinal assessment for lung diseases [85]. Concerning single photon emission computed tomography (SPECT) scanning, although gamma-scintigraphy with ^111^In- labelled granulocytes has been used to monitor migration of polynuclear cells into the lungs [86, 87], its metabolic activation and phenotype remain inaccessible with this nuclear exam [88].

**Figure 1:**
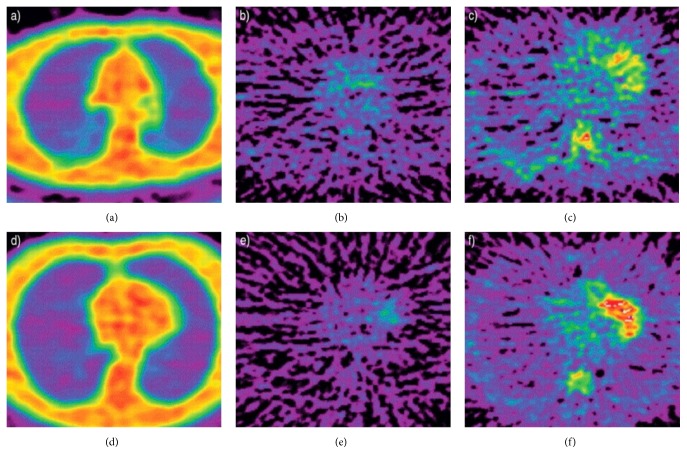
Transaxial thoracic images for transmission (a and d), ^18^F-FDG emission (b and e) and ^11^C-PK11195 (c and f) in a normal subject (a–c), and a patient with chronic obstructive pulmonary disease (d–f) from Jones et al. (2003) [100].

A major concern for the meaning of this TSPO PET imaging approach is the multicellular expression of TSPO in the human lungs. In addition to resident alveolar macrophages, submucosal glands, pneumocytes, and epithelial lining of the airways expression of TSPO have been reported [89, 90] but not neutrophilic expression [91]. Asthma and chronic obstructive pulmonary disease (COPD) are two obstructive respiratory conditions where an important accumulation of inflammatory cells in the respiratory tract takes place and leads to chronic symptoms with periodic acute exacerbations of both asthma and COPD. The pathophysiology of these lung inflammatory disorders is distinct, involving rather excessive formation of eosinophils, mast cells, and CD4^+^ T cells in airways of asthmatics whereas, in COPD, typically neutrophils, macrophages, and CD8^+^ T cells infiltrate the respiratory tract [92]. TSPO seems to play a pathogenic role in asthma and COPD. Indeed, TSPO exhibited downregulated expression in patients with acute exacerbations of COPD [93] and a protein-protein interaction network analysis revealed that TSPO could be implicated in development and/or progression of asthma in children [94]. TSPO is involved in various complex cellular processes such as intracellular transportation, mitophagy, and apoptosis [95–97]. Among them, mitochondrial dysregulation and especially mitophagy represent a key player of signaling pathways relevant to remodeling in chronic remodeling lung pathologies like asthma [98] and COPD [99]. Quantitative analysis of the acquired emission TSPO PET data is in consonance with these findings where mean tracer uptake, objectified by plateau tissue/plasma, was higher in COPD patients and asthmatics than in healthy volunteers. Moreover, pulmonary TSPO PET imaging used in combination with ^18^F-FDG indicated that metabolic activation of inflammatory cells was higher in COPD patients than in chronic asthmatics [100]. Furthermore, cigarette smoke exposure, which is the leading cause of COPD, altered directly TSPO expression, paving the way for lung cancerization [101]. It should be noted that no obvious differences are seen between patients and healthy subject in the emission images for either ^18^FDG or ^11^C-PK11195, due to the low density of the lung (Figure 1). Significant differences are only revealed by quantitative analysis of the acquired emission data [100].

TSPO PET imaging has been used to assess, in humans, macrophages involvement in interstitial lung diseases [102]. Surprisingly, Branley et al. [85] demonstrated that TSPO expression is reduced in these lung inflammatory disorders. Given the fact that patients with interstitial lung disease showed an accumulation of intrapulmonary macrophages [103] and experimental models of pulmonary inflammation revealed to be associated with TSPO overexpression [104], authors hypothesized that macrophages phenotypically differed in these patients (i.e., macrophage with reduced TSPO expression) [102]. In parallel to the relationship between lung density and disease activity (i.e., between controls, patients treated, and drug-naive patients), ^11^C-PK11195 binding would tend to decline according to the disease burden in fibrosing alveolitis due to systemic sclerosis (FASSc) patients [85]. As a result of these achievements, TSPO PET imaging could be a decision-support exam to initiate immunosuppressive treatment in FASSc.

TSPO PET imaging did not provide unequivocal results [85, 100] and in front of some limitations (e.g., multicellular expression of TSPO in human lungs [89, 90], high variability in radiotracer uptake between COPD and asthmatic patients, and wide overlap between patients and controls [100]), TSPO did not seem to be a timely biomarker to diagnose and even less to improve our knowledge in lung disease pathophysiology. In addition, the apparent TSPO downregulation in interstitial lung diseases [85] and acute exacerbations of BPCO [93] should not be construed as absolute finding; indeed TSPO expression is a dynamic process and highly context-dependent, which is probably integrated in a time-dependent fashion (acute versus chronic injury).

### 3.4. Inflammatory Vasculopathies (See Table 4)

**Figure 2:**
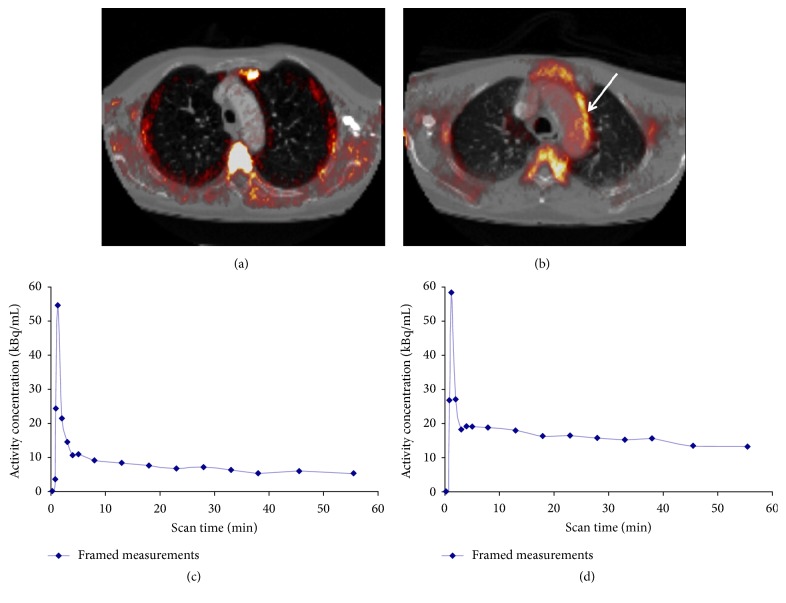
^11^C-PK11195 PET/CT angiography of asymptomatic (a) and symptomatic (b) patient with vasculitis. Each patient is shown in transverse view. Arrow indicates inflamed region of aortic arch. Respective time-activity curves (corrected for radioactive decay) derived from aortic vessel wall (c and d) from Lamare et al. (2010) [110].

TSPO is involved in pathophysiology of various cardiovascular diseases, including arrhythmia, myocardial infarction, cardiac hypertrophy, atherosclerosis, myocarditis, and large vessel vasculitis (for review see Qi et al. 2012 [105]). Among these, large vessel vasculitis represents a heterogeneous group of complex disorders (e.g., giant cell arteritis, Takayasu's arteritis) in which chronic inflammatory lesions of the blood vessels are characterized by granulomatous pan-arteritis with focal leukocytic infiltration [106]. Typical clinical symptoms include the association of blindness, stroke, claudication according to the vascular territory affected/occluded, fever, night sweats, and arthralgia [107–109]. TSPO molecular imaging using ^11^C-PK11195 provided valuable information such as presence, intensity, and localization of activated macrophages in large vessel vasculitis [36, 110]. In these studies, giant cell arteritis, Takayasu's arteritis, and lupus erythematosus patients fulfilling American College of Rheumatology diagnostic criteria [111] were enrolled. Authors defined active vasculitis as onset within the previous 3 months of any of the symptoms mentioned above and conversely asymptomatic patients' diagnosis based on absence of symptoms of active disease. The fact that TSPO PET signal was quantifiable even in some asymptomatic patients (Figure 2) paves the way of a preemptive therapeutic strategy, that is to say prior the symptoms, whereas the inflammatory process is active [36, 110]. Although only one giant cell arteritis patient was enrolled for a longitudinal assessment of corticosteroids response, TSPO PET imaging could be a promising approach to monitor drug efficacy of immunosuppressive agents currently used and for drug development in vasculitis. Patients with large vessel vasculitis have an increased cardiovascular risk compared to the age-matched healthy population as a consequence of accelerated atherosclerosis [106].

Atherosclerosis is an inflammatory, chronic metabolic disorder of the arterial walls, in which intraplaque inflammation drives the progression and the destabilization of atheromatous lesions [112, 113]. It seems that atherosclerosis starts with the penetration of low-density lipoprotein (LDL) through a damaged endothelial wall, leading to their oxidation. These oxidized LDL particles act as autoantigens presented by macrophages which differentiate into foamy macrophages, promote the inflammation, and cause interleukins secretion [114–116]. According to its function as leader of cholesterol transport and steroidogenesis [97] and its dysregulation induced by smoke exposure [101], TSPO has been targeted in drug development of lipid-lowering therapy and “cardiovascular” anti-inflammatory/antioxidant treatment [117–119]. Autoradiography and immunostaining experiments highlighted that TSPO tracer binding concerned predominantly CD68-positive areas of mice carotid sections [120] and human carotid endarterectomy samples [121, 122] which contained fibrotic and calcified tissue [121]. Recent research by Chinetti-Gbaguidi and colleagues [123] revealed that RANKL (receptor activator of nuclear factor *κ*B (NF-*κ*B) ligand) and MCSF (macrophage colony-stimulating factor), two major mediators of vascular calcification, are dysregulated by IL-4 polarization (i.e., M_1_ to M_2_ polarization), leading macrophages surrounding atherosclerotic plaques to be unable to resorb calcification. Therefore, the lack of association between plaque calcification score and ^11^C-PK11195 target-to-background ratio (TBR) [122] could be explained by a different macrophage phenotype (i.e., M_2_ polarized) which would be undetectable on PET images. Moreover, in the same clinical study TSPO PET imaging of patients with carotid stenosis revealed that ^11^C-PK11195 standardized uptake value (SUV) and TBR were significantly higher in carotid atheromatous lesions of symptomatic patients (i.e., associated with stroke and transient ischemic attacks) compared to asymptomatic individuals. Surprisingly, no difference was found between the severity of carotid stenosis on CT angiography between symptomatic and asymptomatic patients [122]. Most importantly, it appears that activated macrophages, assessed by TSPO PET, were detected at a disturbed flow site located downstream from the region of stenosis [120]. Finally, multimodal imaging using ^11^C-PK11195 PET in combination with CT plaque attenuation offers good diagnostic performances to improve risk stratification in patients with asymptomatic carotid stenosis in order to anticipate cerebrovascular events [122]. Beyond this potential diagnostic role of TSPO PET scanning for atherosclerosis, TSPO might be used as a therapeutic target for atherosclerosis. Indeed, the oxidative stress induced by high fat and high cholesterol atherogenic diet in rats has been associated with a reduction of TSPO-binding density [124].

### 3.5. Rheumatic and Musculoskeletal Disorders (See Table 5)

**Figure 3:**
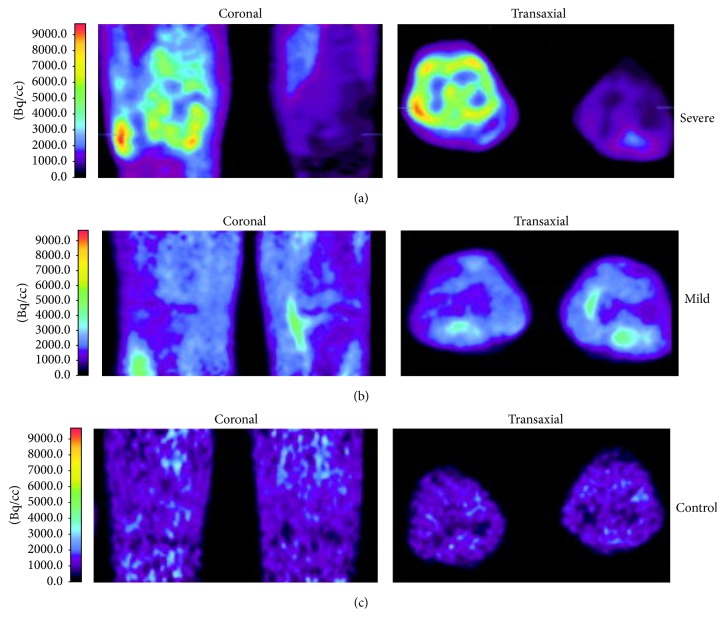
^11^C-PK11195 PET images in coronal and transaxial directions. (a) Images of severe clinical inflammation of the right knee (depicted at the left in both images) and no clinical inflammation of the left knee in a patient with rheumatoid arthritis (RA). (b) Images of mild inflammation of both knee joints in an RA patient. (c) Images of knees without joint disease in a control subject. The different levels of tracer uptake correspond to the colors in the color bar at the left, from van der Laken et al. (2008) [125].

Rheumatoid arthritis (RA) is a chronic autoimmune disease characterized by joint inflammation where the diagnosis is based on joint involvement, duration of symptoms, serology (i.e., rheumatoid factor, anticitrullinated protein antibody), and biology (i.e., erythrocyte sedimentation rate, C-reactive protein) [126, 127]. TSPO PET imaging in various animal models of noninfectious arthritis supported the fact that nuclear-based approach provides quickly information on biological functions even before anatomical alterations of bone and cartilage [128–130]. TSPO PET imaging appears to be a promising approach to follow early events in the pathophysiology of RA, suggesting that a precocity medical care should be feasible even before the structural alterations, especially as the uninflamed knee joints of RA patients showed a significant greater TSPO tracer uptake than in healthy controls [125]. Moreover, association analysis showed a good correlation between tracer binding in joints and clinical synovial swelling or macrophage infiltration in synovial tissue in preclinical [130] and clinical study [125].  Figure 3 illustrates these findings. For imaging of RA, the key limitation of TSPO PET scanning is the tracer uptake in the inflamed synovium linked to binding in surrounding bone and bone marrow (e.g., periarticular bone of joints binding), keeping from quantifying accurately uptake in the synovium [129]. Nevertheless, this problem appears to be minimized when ^18^F-DPA tracers have been used in rat model of rheumatoid arthritis (i.e., better knee-to-bone ratios), compared to ^11^C-PK11195 [129].

## 4. TSPO PET Imaging: Towards a Clinical Application for Pathologies with Both Central and Peripheral Inflammatory Component?

TSPO PET imaging has to date been used mainly to assess microglial activation in various neurologic diseases ranging from neurodegenerative disorders such as Huntington's disease [131] and Alzheimer's disease [132] to stroke [133] and psychiatric conditions like schizophrenia [134]. The promising results of TSPO PET imaging to diagnose and characterize some PSIDs and especially atherosclerosis [122] lead us to think that, as in some central nervous system disorders such as multiple sclerosis and amyotrophic lateral sclerosis [39], a clinical application of TSPO as a biomarker of inflammation is possible. Nevertheless, as with neurologic disorders some limitations must be taken like spillover and partial volume effect (because of proximity of the blood pool and limited thickness of the arterial wall), a multicellular expression making mathematic model to quantify free and specific ligand binding more complex. Furthermore, in contrast to central conditions where tracer's radiolabeled metabolites are not sufficiently lipophilic to produce background noises, in PSIDs the pharmacokinetic of radiolabeled metabolites leads to hinder TSPO signal quantification. This is especially a limitation for IBDs where tracer elimination by the upper digestive tract (e.g., ^18^F-DPA-714) compromised TSPO quantification [25].

The strength of TSPO PET imaging could rely on the ability to detect inflammatory changes in pathologies which have central and peripheral expression, for instance, to evaluate the relationship between neuroinflammation induced by stroke and TSPO expression of atherosclerotic plaques in patients with carotid stenosis. Indeed, it allows characterizing inflammation and establishing if interplay occurred between microglial activation and peripheral macrophages. In this sense, surprising findings have been found in liver fibrosis where TSPO are not overexpressed in patients with hepatic encephalopathy [135]. Likewise, in a preclinical study, inflammation in both the gut and the brain of rats with chemically induced colitis was observed by* ex vivo* biodistribution but these effects could not be detected by ^11^C-PBR28 PET imaging which was likely due to insufficient resolution of the micro-PET camera [136]. Besides PSIDs, infectious diseases where TSPO PET imaging has already been investigated such as HIV infection [137] or sepsis [138] could benefit from this approach in order to know if central and peripheral inflammation is a continuum or acts independently.

## 5. Conclusion

The pathophysiologic involvement of TSPO in PSIDs is well-documented especially in cardiovascular conditions [105] at the opposite of microglial activation in neurologic disorders which remains controversial. Limitations of TSPO PET imaging in PSIDs concern the large expression of TSPO in peripheral tissues whereas, in central nervous system, TSPO expression is low in healthy brain [39]. A body of evidence gives a M1-phenotype biomarker status of microglial TSPO expression [139]. In line with these findings, the fact that TSPO PET imaging did not highlight significant signal in some PSIDs (e.g., atherosclerosis [122], interstitial lung disease [85]), where macrophage activation is now well-documented, seems to confirm that, also in peripheral disorders, TSPO may rather to be associated with harmful inflammatory state than regenerative environment. Nevertheless these in vitro findings need to be* in vivo* translated [139].

## Figures and Tables

**Table 1:** 

Applications	Population/Animal models	Radioligand	Main findings	Ref.
Preclinical	(i) TSPO PET on day 8 after DSS- and TNBS-induced IBD in rats (ii) 6 rats were followed up over 22 days	^18^F-DPA-714	(i) Significantly increased tracer uptake in the rat colon in both groups, DSS and TNBS, compared to controls (ii) Increased tracer binding until day 8/10 then tracer uptake decreased slowly	Bernards et al. (2014) [25]

DSS, dextran sodium sulfate; IBD, inflammatory bowel disease; TNBS, trinitrobenzenesulfonic acid.

**Table 2:** 

Applications	Population/animal models	Radioligand	Main findings	Ref.
Preclinical	6 mice per each group 2-, 4-, and 8-week MCD-fed mice versus control mice	^18^F-FEDAC	In 4-week MCD livers, higher binding of tracer was observed at some *loci* in the right lobe compared to the left liver lobe Significantly increased uptake ratios of liver to blood in 2-, 4-, and 8-week MCD-fed mice, compared to controls Strong correlation between uptake ratio of tracer binding and NAFLD activity score was found during NAFLD progression	Xie et al. (2012) [79]

Preclinical	4 rats per each group after 2, 4, 6, and 8 weeks of CCl_4_ treatment versus control rats	^18^F-FEDAC	Significantly higher liver tracer SUV in all treatment groups, compared to controls Tracer binding positively correlated with CCl_4_ treatment duration and severity of liver damage	Hatori et al. (2015) [80]

MCD, methionine and choline deficient; NAFLD, nonalcoholic fatty liver disease; SUV, standardized uptake value.

**Table 3:** 

Applications	Population/animal models	Radioligand	Main findings	Ref.
Preclinical	Unilateral instillation of fibrogenic (microcrystalline SiO_2_, *n* = 12 rabbits), nonfibrogenic (amorphous SiO_2_, *n* = 10 rabbits) 5 *μ*m particles into rabbit lungs	^11^C-PK11195	Significantly increased tracer tissue/plasma ratios from day 3 after instillation in the challenged region, in both silica models, and remaining at least 2 weeks In the amorphous silica model, an extrapulmonary TSPO PET signal appeared at 6 days In the microcrystalline silica model, this extrapulmonary signal is delayed until 3 weeks after instillation	Jones et al. (2002) [91]

Clinical	6 COPD patients, 6 chronic asthmatics, and 5 HC	^11^C-PK11195	Mean tracer uptake was higher in 4/6 COPD patients and 3/6 asthmatics than the maximum value in HC No statistical correlation was found between binding tracer and either the magnitude of the ^18^F-FDG signal or the severity of the disease	Jones et al. (2003) [100]

Clinical	15 FASSc (10 drug-naive & 5 with immunosuppressive drugs) patients versus 7 HC	^11^C-PK11195	Tracer lung uptake was decreased in FASSc patients, compared to normal controls Strong negative correlation between tissue radioligand uptake and lung density in FASSc patients Pulmonary tracer binding showed a nonsignificant but progressive decrease from normal in FASSc drug-naive patients to those patients immunosuppressive-treated No significant correlation between tracer uptake and pulmonary function data	Branley et al. (2008) [85]

COPD, chronic obstructive pulmonary disease; FASSc, fibrosing alveolitis due to systemic sclerosis; HC, healthy control.

**Table 4:** 

Applications	Population/animal models	Radioligand	Main findings	Ref.
Vasculitis				

Clinical	(i) 15 patients with large vessel vasculitis (5 TA, 4 GCA, & 6 SLE) (ii) 1 GCA patient followed-up after 20-week course of oral CS	^11^C-PK11195	(i) 3/5 patients whose tracer binding was found at the level of the aorta; maximal tracer uptake coincided with minimal calcification Tracer TBR was significantly higher in symptomatic, compared to asymptomatic patients (ii) Tracer uptake was markedly reduced in the wall of the aortic arch after CS treatment The reduction in radioligand binding was paralleled by a distinct improvement in symptoms and a decrease in his serum inflammatory markers	Pugliese et al. (2010) [36]

Clinical	7 patients (3 symptomatic & 4 asymptomatic) with large vessel vasculitis	^11^C-PK11195	*V*_*T*_ was significantly greater in symptomatic than in asymptomatic patients	Lamare et al. (2010) [110]

Atherosclerosis				

Preclinical	Constrictive cuff was placed on the right carotid artery of 6 mice	^18^F-FEDAA1106	Significantly increased tracer binding in the right carotid artery compared to left (unmanipulated) carotid artery TAC exhibited a significantly greater increase in binding at the downstream region when compared to the cuffed or upstream regions at 60 minutes after injection	Cuhlmann et al. (2014) [120]

Preclinical	10 atherosclerotic mice (LDLR^−/−^ ApoB^100/100^) versus 9 healthy mice	^18^F-FEMPA	There was visually detectable tracer uptake that colocalized with the aortic arch, but not significantly different between the animal groups Radioligand SUV was significantly higher in atherosclerotic aortas in muted mice than in healthy control mice	Hellberg et al. (2017) [121]

Clinical	32 patients with carotid stenosis (9 symptomatic & 27 asymptomatic)	^11^C-PK11195	Tracer SUV and TBR were higher in carotid plaques of symptomatic than asymptomatic patients No significant correlation between radioligand TBR and plaque calcification ROC analysis of tracer TBR to identity patients with CVE showed sensitivity of 78%, specificity of 74%, NPV of 91%, and PPV of 50%	Gaemperli et al. (2012) [122]

CS, corticosteroids; CVE, cerebrovascular events; GCA, giant cell arteritis; HC, healthy control; NPV, negative predictive value; PPV, positive predictive value; ROC, receiver operating characteristic; SLE, systemic lupus erythematosus; SUV, standardized uptake value; TA, Takayasu's arteritis; TAC, time-activity curves; TBR, target-to-background ratio; *V*_*T*_, total volume of distribution.

**Table 5:** 

Applications	Population/animal models	Radioligand	Main findings	Ref.
Rheumatic diseases				

Preclinical	6 rats were injected with 1% carrageenan solution into the paw 8 genetically susceptible rats were injected with inactivated *M. butyricum *into the nail	^11^C-PBR28	Significantly higher tracer SUV_peak_ in carrageenan-treated paws compared to paired contralateral controls Increased radioligand mean AUC_SUV_ of 60-minute time-activity data was found at the root of the tail, sacroiliac joints, and knees, compared to controls	Shao et al. (2013) [128]

Preclinical	18 rats (*n* = 7, 6, 5 for each tracer, resp.) were injected with mBSA to induce knee arthritis versus 5 untreated rats	^11^C-DPA-713/^18^F-DPA-714/^11^C-PK11195	All three TSPO tracers clearly accumulated in arthritic knees Mean absolute SUV of the three radioligands was markedly higher than in contralateral knees	Gent et al. (2014) [129]

Preclinical	11 rats were injected with inactivated *M. tuberculosis* into the tail versus 6 rats nontreated	^18^F-DPA-714	Increased tracer binding in ankles of treated rat, compared to control rat at 20 days The mean radioligand uptake value in treated animals was more than twice that of nontreated animals Association analysis exhibited a good relation between tracer uptake and the joint swelling	Pottier et al. (2014) [130]

Clinical	10 RA patients with active arthritis versus 8 HC	^11^C-PK11195	The mean *V*_*T*_ and the mean SUV ratios were significantly higher in knees with score severe synovial swelling score than those in mild and absent synovial swelling SUV ratios in the contralateral uninflamed knee joints of RA patients were 50% higher than those in uninflamed knee joints of healthy volunteers	van der Laken et al. (2008) [125]

Musculoskeletal disorders				

Preclinical	Mouse were injected with oil of turpentine into the left thigh muscle and then followed-up for 26 days after injection	^18^F-DPA-714	The inflammatory muscles showed significantly increased local accumulation of tracer compared with collateral muscle The uptake peaked on day 6 and then dropped slowly along with the time till day 26, which was still higher than that in collateral muscle	Wu et al. (2014) [140]

AUC, area under the curve; HC, healthy controls; mBSA, methylated bovine serum albumin; RA, rheumatoid arthritis; SUV, standardized uptake value; *V*_*T*_, total volume of distribution.
